# Role of SES on the association between childhood parental death and adulthood suicidal ideation: a mediation analysis using longitudinal dataset in South Korea

**DOI:** 10.1186/s12888-021-03146-w

**Published:** 2021-03-23

**Authors:** Jaehong Yoon, Ja Young Kim, Ji-Hwan Kim, Seung-Sup Kim

**Affiliations:** 1 Department of Public Health Sciences, Graduate School of Korea University, Seoul, South Korea; 2 Gyeonggi Public Health Policy Institute, Seongnam-si, Gyeonggi-do, Republic of Korea; 3 Interdisciplinary Program in Precision Public Health, Korea University, Seoul, South Korea; 4 Department of Social & Behavioral Sciences, Harvard T.H. Chan School of Public Health, Boston, MA USA

**Keywords:** Childhood adversities, Mediation analysis, Suicide, Mental health, Life-course

## Abstract

**Background:**

We sought to examine the association between childhood experience of parental death (CEPD) and adulthood suicidal ideation, and the mediating role of adulthood SES in the association.

**Methods:**

We analyzed a nationally representative dataset of 8609 adults from the Korea Welfare Panel Study, which is a longitudinal cohort dataset in South Korea. CEPD was measured using a question: “During your childhood (0-17 years old), have you experienced the death of parents?” We classified responses of CEPD during 2006–2011 into ‘yes,’ and the others into ‘no.’ Suicidal ideation over the past year was assessed annually during 2012–2019. As a potential mediator, adulthood educational attainment and household income in 2011 were included in the analysis. Logistic regression was applied to examine the association of CEPD with adulthood suicidal ideation across age groups (early adulthood, 19–39 years old; middle adulthood, 40–59 years old; late adulthood, ≥60 years old), after excluding people who reported lifetime suicidal ideation in 2011. Causal mediation analysis using a parametric regression model was applied to examine the mediating role of adulthood SES in the association between CEPD and adulthood suicidal ideation.

**Results:**

After adjusting for potential confounders including childhood SES, CEPD was significantly associated with adulthood suicidal ideation among the late adulthood group (OR: 1.43; 95% CI: 1.13–1.81), while the association was not statistically significant among the early; and middle adulthood groups. In mediation analysis of adulthood household income, both indirect association (OR^NIE^: 1.05; 95% CI: 1.02–1.09) and direct association (OR^NDE^: 1.37; 95% CI: 1.09–1.73) were statistically significant among the late adulthood group. In the mediation analysis of adulthood education attainment among the late adulthood, only a direct association was statistically significant (OR^NDE^: 1.43; 95% CI: 1.14–1.80).

**Conclusions:**

These results suggest that CEPD could be a risk factor for adulthood suicidal ideation. Furthermore, the findings imply that income security policy might be necessary to reduce suicide among the late adulthood group.

**Supplementary Information:**

The online version contains supplementary material available at 10.1186/s12888-021-03146-w.

## Introduction

**Fig. 1:**
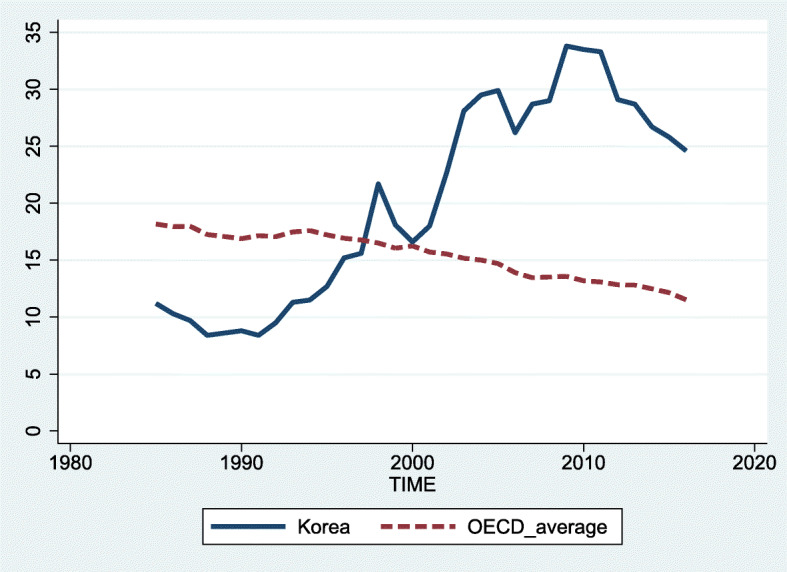
Trends in suicidal death in South Korea compared to OECD global average

Suicide is a worldwide public health concern, with an annual global age-standardized suicide rate of 10.5 deaths per 100,000 in 2016 [1]. In South Korea (hereafter Korea), suicide is a critical issue. The age-standardized suicide rate of Korea was 20.2 per 100,000 persons in 2016, which was twice as high as those of the WHO average. In addition, the suicide rate in Korea dramatically increased from 11.2 deaths per 100,000 persons in 1985 to 24.6 deaths per 100,000 persons in 2016, while the OECD global average suicide rate decreased from 18.2 deaths per 100,000 persons in 1985 to 11.5 deaths per 100,000 persons in 2016 [2] (Fig. 1).

According to cumulative inequality theory, childhood is a critical period to shape adulthood life conditions, and so adverse experiences during this period could contribute to health inequality in adulthood [3, 4]. Because childhood conditions could reflect the intergenerational transmission of genetic and socioeconomic factors and play a role in shaping future lifestyles and resources, it can determine long-term health conditions. Childhood experience of parental death (CEPD) is a major stressful event [5]. Growing evidence suggests that CEPD might be an important risk factor for adulthood mental health issues, including suicidal behaviors [6–8]. However, previous studies have reported inconsistent associations between CEPD and adulthood mental health. A study examining whether childhood trauma and parental death are associated with adulthood suicidal risk among 1396 adults in Korea showed that CEPD is not significantly related to current suicidality risk [9]. Another study in Korea showed that male adults who experienced maternal death before 10 years of age were more likely to report lifetime suicide attempts than those who did not experience maternal death [10].

Further, there are inconsistent reports of the association between CEPD and adulthood mental health across age groups. A cohort study of 663,729 people in the United States reported that early-life parental death is not significantly associated with a higher risk of adulthood suicide among individuals ≥50 years old [11]. However, a study in Korea using a nationally representative sample showed a significant relationship of CEPD with the prevalence of adult depressive symptoms among individuals ≥60 years old [12]. This inconsistency among the late adulthood population could occur because of the different economic situations across countries when the elderly population was in childhood. For example, since Korea is a country where rapid economic growth began around the 1970s, the poverty rate in Korea was high when the Korean elderly population was in childhood.

Finally, people with CEPD would be less likely to attain higher socioeconomic status (SES) than people without CEPD [3, 4]. However, few studies have examined the mediating role of adulthood SES on the association between CEPD and mental health problems in adulthood. A cross-sectional study of 814 adults in Korea found that educational attainment may play a mediating role in the association between CEPD and adulthood depressive symptoms [13]. Also, it would be necessary to measure the incidence of mental health problems rather than the experience during the lifetime when conducting mediation analysis in a life-course perspective. Although lifetime measurement of mental health problems might not ascertain temporal order between mediator and outcome, few studies utilized the new-onset mental health problems to assess the mediating role of adulthood SES in the association between CEPD and adulthood mental health.

To fill these knowledge gaps, we sought to address the following questions using a nationally representative dataset of Korean adults: (a) Is CEPD associated with the incidence of adulthood suicidal ideation? Does the association vary by age groups? (b) Is the association mediated by adulthood SES?

## Methods

### Study population

This study analyzed a nationally representative longitudinal dataset from the Korea Welfare Panel Study (KOWEPS), which launched in 2006. A total of 7072 households (18,856 respondents) who were selected using two-stage stratified cluster sampling participated at the 1st wave of KOWEPS which annually added branched households originating from the 1st wave households. To date, all of the KOWEPS data are publicly available online (www.koweps.re.kr), and we utilized data from waves 1–14.

Our analysis included a total of 8609 adults who were ≥ 19 years old, who have never experienced lifetime suicidal ideation at the 6th wave of the survey, and who did not have any missing information on experience of suicidal ideation, CEPD, and covariates. Because KOWEPS is a publicly available dataset, this study received Institutional Review Board (IRB) exemption from the Office of Human Research Administration at Korea University.

### Measures

#### Suicidal ideation

KOWEPS has annually measured suicidal ideation over the past year from waves 7–14 (2012–2019) of the survey. Suicidal ideation was measured with the question: “Have you ever seriously thought about dying by suicide within the past 12 months?” Participants could answer yes or no. After combining the responses from each wave, we defined those who experienced suicidal thoughts at least once during 2012–2019 as having experienced suicidal ideation.

#### Childhood experience of parental death (CEPD)

CEPD was assessed when participants first joined the study between wave 1 (2006) and 6 (2011), using the following question: “During your childhood (0–17 years old), have you experienced death of parents?” Participants answered yes or no. We classified responses of CEPD during 2006–2011 into “yes”, and the others into “no.”

#### Adulthood education and income

As mediators, we utilized adulthood education level and household income measured at the 6th wave. We differently categorized adulthood education attainment across age groups because the meaning of high school graduates in early (19–39 years old) and middle adulthood (40–59 years old) might be different from those in late adulthood (≥60 years old). In early and middle adulthood, participants whose educational level was college graduate or more were classified into ‘high’ education attainment group (‘low’: high school graduate or lower); in the late adulthood group, those whose educational level was a high school graduate or more were defined as ‘high’ education attainment group (‘low’: middle school graduate or lower). Equivalized household income was calculated by dividing the summed household income by the square root of the number of household members. In the analysis, we transformed equivalized household income into log scale.

#### Potential confounders

As potential confounders that may be associated with both adulthood suicidal ideation and CEPD, we included age, sex, and childhood SES (i.e., education level of each parent and occupation of each parent during childhood) measured at 6th wave in the analysis. Age was classified into 6 groups: 19–29, 30–39, 40–49, 50–59, 60–69, and ≥ 70 years old. The education level of each parent was categorized into 4 groups: no education, elementary school graduate, junior high school graduate, high school graduate or more. Occupation of each parent was measured in accordance with the International Standard Classification of Occupations and was classified as non-manual labor, manual labor, or homemaker/other. A detailed explanation for the classification of occupation is provided in previous KOWEPS literature [12, 14].

### Data analysis

Descriptive analyses were conducted to assess the distributions of study population across key covariates and the relationship of covariates with CEPD and suicidal ideation by applying Rao-Scott design-adjusted χ2 test and t-test with sample weights.

We conducted three steps to examine the relationship of CEPD with suicidal ideation and the role of adulthood SES on the association without applying the sample weights. First, we collapsed age categories into three groups because adulthood SES might represent different meanings at different life stages: early adulthood (19–39 years old), middle adulthood (40–59 years old), and late adulthood (≥60 years old). Before conducting an age-stratified analysis, interaction between CEPD and age were assessed in both multiplicative and additive scale. Second, we examined the age-specific association between CEPD and incidence of suicidal ideation by applying logistic regression after excluding people who reported lifetime experience of suicidal ideation at the 6th wave. Since the suicidal ideation of adults from the same household was likely to be correlated with each other, robust standard error was estimated by using adulthood household identifier (the number of households = 4618). Third, we applied a parametric regression model using ‘paramed’ command in Stata to examine the mediating role of adulthood SES on the association [13]. A causal mediation analysis using a parametric regression model extends traditional mediation analysis by allowing the exposure–mediator interactions, and, therefore, it estimates natural indirect effect (NIE), natural direct effect (NDE), controlled direct effect (CDE), and marginal total effect (MTE). Results were presented as odds ratios (ORs) with 95% confidence intervals (CIs). MTE means the total effect between CEPD and adulthood suicidal ideation, NIE is the effect of CEPD to adulthood suicidal ideation through adulthood SES, and NDE is the effect of CEPD to adulthood suicidal ideation not through adulthood SES. CDE is the direct effect when a mediator is fixed to a specific value, which is reported in the presence of exposure-mediator interaction. Because the interaction between CEPD and adulthood SES was not significant, we did not report CDE. All analyses were done using Stata/SE version 13.0 (Stata Corp., College Station, TX, USA).

## Results

Table 1 presents the distribution of the study population and the incidence of suicidal ideation by covariates after excluding people with lifetime suicidal ideation at baseline. The estimated total number of adults at baseline was 29,475,015. The weighted proportion of CEPD and incidence of suicidal ideation was respectively 13.1% (95% CI: 12.3–14.0), and 7.2% (95% CI: 6.5–7.9). The incidence of suicidal ideation was significantly higher among older individuals, and people whose parents had lower education. CEPD was more common among respondents whose parents had no education, and whose parents were in manual occupation. As shown in Table 2, suicidal ideation was more frequent among respondents who had a low education level.

**Table 1:** Weighted percentage of study population, childhood parental death, and incidence of adulthood suicidal ideation during 2012–2019 by key covariates (*N* = 29,475,015)

Variable	Total	Childhood experience of parental death		Incidence of suicidal ideation	
	Weighted percentage of total	% (95% CI)	*P*-value^a^	% (95% CI)	*P*-value^b^
Total	100.0	13.1 (12.3–14.0)		7.2 (6.5–7.9)	
Gender			0.100		0.050
Male	48.9	13.8 (12.6–15.2)		6.5 (5.6–7.5)	
Female	51.1	12.4 (11.3–13.6)		7.8 (6.9–8.8)	
Age			< 0.001		< 0.001
19–29	19.2	4.4 (3.2–6.1)		4.2 (2.9–6.0)	
30–39	22.1	9.7 (8.1–11.5)		5.9 (4.7–7.3)	
40–49	22.6	14.2 (12.2–16.4)		6.8 (5.5–8.5)	
50–59	17.4	15.6 (13.4–18.1)		8.2 (6.6–1.2)	
60–69	10.2	20.6 (18.0–23.3)		10.0 (8.3–11.9)	
≥ 70	8.4	24.7 (22.3–27.3)		12.6 (10.9–14.6)	
Education of father			< 0.001		< 0.001
No education	19.2	21.5 (19.5–23.7)		9.9 (8.7–11.4)	
Elementary school graduate	32.3	15.8 (14.2–17.6)		8.2 (7.1–9.6)	
Junior high school graduate	14.4	10.5 (8.4–12.9)		6.3 (4.8–8.3)	
High school graduate or more	34.1	6.7 (5.7–8.2)		4.9 (3.9–6.2)	
Education of mother			< 0.001		< 0.001
No education	30.5	20.4 (18.8–22.2)		9.9 (8.8–11.2)	
Elementary school graduate	34.3	13.5 (12.0–15.1)		7.4 (6.3–8.7)	
Junior high school graduate	14.3	7.8 (5.9–10.2)		5.6 (4.2–7.4)	
High school graduate or more	20.9	5.4 (4.1–7.1)		3.9 (2.7–5.5)	
Occupation of father			< 0.001		0.086
Non-manual	19.8	8.8 (7.2–10.8)		5.7 (4.4–7.2)	
Manual	78.2	14.2 (13.3–15.3)		7.6 (6.9–8.4)	
Others	2.0	10.9 (7.0–16.6)		5.7 (2.5–12.2)	
Occupation of mother			< 0.001		0.339
Non-manual	1.9	1.6 (0.4–5.9)		4.4 (1.4–12.9)	
Manual	61.3	16.1 (14.9–17.3)		7.6 (6.8–8.5)	
Others	0.2	16.6 (5.7–39.6)		7.5 (1.7–28.2)	
Homemaker	36.6	8.7 (7.5–10.0)		6.5 (5.5–7.8)	

^a^: *P*-value of the Rao-Scott design-adjusted chi-square test comparing the CEPD across the different groups

^b^: *P*-value of the Rao-Scott design-adjusted chi-square test comparing the incidence of suicidal ideation across the different groups

**Table 2:** Weighted distribution of mediators (adulthood education attainment and adulthood household income), and incidence of adulthood suicidal ideation in Korea

	Suicidal ideation in 2012–2019								
	Early adulthood: 19–39 years old (*N* = 12,171,989)			Middle adulthood: 40–59 years old (*N* = 11,802,078)			Late adulthood: ≥60 years old (*N* = 5,500,948)		
	Weighted percentage of total	Incidence of suicidal ideation		Weighted percentage of total	Incidence of suicidal ideation		Weighted percentage of total	Incidence of suicidal ideation	
		% (95% CI)	*P*-value		% (95% CI)	*P*-value		% (95% CI)	*P*-value
Education attainment			0.004^a^			0.027 ^a^			0.021 ^a^
Low	30.8	7.2 (5.6–9.2)		67.9	8.5 (7.2–9.9)		70.5	12.2 (10.8–13.8)	
High^d^	69.2	4.1 (3.1–5.5)		32.1	5.3 (3.6–7.8)		29.5	8.7 (6.6–11.3)	
Household income^e^	54.5 (0.3)^c^	50.5 (1.3)^c^	0.002^b^	54.55 (0.5)^c^	48.1 (1.3)^c^	< 0.001^b^	41.7 (0.4)^c^	36.3 (0.7)^c^	< 0.001^b^

^a^: *P*-value of the Rao-Scott design-adjusted chi-square test comparing the incidence of suicidal ideation across the different groups

^b^: *P*-value of the t-test comparing the incidence of adulthood suicidal ideation, and mean of the household income

^c^: Mean (standard deviation)

d: The early and middle adulthood groups: high school graduate or lower (low), and college graduate or more (high); the late adulthood group: middle school graduate or lower (low), and high school graduate or more (high)

^e^: Equivalized household income with logarithmic transformation

Although interaction between CEPD and age group were not statistically significant on a multiplicative scale, the interaction between CEPD and age (middle adulthood vs. late adulthood) was marginally significant (*p*-value: 0.050) on an additive scale. Therefore, we examined the association between CEPD and adulthood suicidal ideation after being stratified by age groups. After adjusting for potential confounders including childhood SES, adulthood incidence of suicidal ideation was associated with CEPD among individuals in late adulthood (OR: 1.43; 95% CI: 1.13–1.81), while the relationship was not statistically significant among individuals in early and middle adulthood (Table 3). When we additionally adjusted for adulthood SES (i.e., education, household income), people who had CEPD were more likely to report adulthood suicidal ideation among the late adulthood group (OR: 1.39; 95% CI: 1.09–1.76), but these association were not statistically significant among early and middle adulthood group (Table 4).

**Table 3:** Association between CEPD and incidence of adulthood suicidal ideation in South Korea (*N* = 8609)

CEPD	Suicidal ideation in 2012–2019											
	Total *N*	Early adulthood: 19–39 years old (*N* = 2614)			Total *N*	Middle adulthood: 40–59 years old (*N* = 2788)			Total *N*	Late adulthood: ≥60 years old (*N* = 3207)		
		*N* (%)	OR	95% CI		*N* (%)	OR	95% CI		*N* (%)	OR	95% CI
No	2408	135 (5.6)	1.00	Referent	2375	187 (7.9)	1.00	Referent	2444	293 (12.0)	1.00	Referent
Yes	206	15 (7.3)	1.23	(0.70–2.16)	413	35 (8.5)	1.05	(0.72–1.53)	763	124 (16.3)	1.43**	(1.13–1.81)

^*^P < 0.05; ^**^P < 0.01; ^***^p < 0.001

Adjusted for age, sex, education level of father and mother, and occupation of father and mother

**Table 4:** Association between CEPD and incidence of adulthood suicidal ideation after adjusting for adulthood socio-economic status in South Korea (*N* = 8609)

CEPD	Suicidal ideation in 2012–2019											
	Total *N*	Early adulthood: 19–39 years old (*N* = 2614)			Total *N*	Middle adulthood: 40–59 years old (*N* = 2788)			Total *N*	Late adulthood: ≥60 years old (*N* = 3207)		
		*N* (%)	OR	95% CI		*N* (%)	OR	95% CI		*N* (%)	OR	95% CI
No	2408	135 (5.6)	1.00	Referent	2375	187 (7.9)	1.00	Referent	2444	293 (12.0)	1.00	Referent
Yes	206	15 (7.3)	1.11	(0.63–1.97)	413	35 (8.5)	1.03	(0.70–1.50)	763	124 (16.3)	1.39**	(1.09–1.76)

^*^P < 0.05; ^**^P < 0.01; ^***^p < 0.001

Adjusted for age, sex, education level of father and mother, occupation of father and mother, education attainment, and household income

We conducted the mediation analysis only in the late adulthood group, because the relationship of CEPD with adulthood suicidal ideation was not statistically significant among the early and middle adulthood group. Table 5 shows NDE, NIE, and MTE of CEPD on adulthood suicidal ideation among the late adulthood group. When adulthood education was considered as a mediator, CEPD was associated with suicidal ideation in late adulthood (OR^MTE^: 1.43; 95% CI: 1.02–2.00). Decomposition of total effects indicated that only NDE was significant (OR^NDE^: 1.43; 95% CI: 1.14–1.80) in late adulthood. In the analysis with adulthood income as a mediator, CEPD was related to adulthood suicidal ideation (OR^MTE^: 1.44; 95% CI: 1.15–1.82). Decomposition of total effects indicated statistically significant NDE (OR^NDE^: 1.37; 95% CI: 1.09–1.73) and NIE (OR^NIE^: 1.05; 95% CI: 1.02–1.09) in late adulthood. Also, we observed consistent results in the sub-analysis, which examined the association between CEPD and adulthood suicidal ideation when we included people who had a lifetime experience of suicidal ideation in the analyses (Supplement 1, 2, 3 in additional file 1).

**Table 5:** The natural direct effects (NDE), natural indirect effects (NIE), and marginal total effects (MTE) expressed as risk ratios (ORs) of CEPD on incidence of adulthood suicidal ideation among the late adulthood group in South Korea (*N* = 3207)

CEPD	Suicidal ideation in 2012–2019					
	NDE		NIE		MTE	
	OR	95% CI	OR	95% CI	OR	95% CI
Adulthood education attainment^a^	1.43**	(1.14–1.80)	1.00	(0.78–1.28)	1.43*	(1.02–2.00)
Adulthood household income^b^	1.37**	(1.09–1.73)	1.05**	(1.02–1.09)	1.44**	(1.15–1.82)

^*^P < 0.05; **P < 0.01; ^***^p < 0.001

Adjusted for age, sex, education level of father and mother, and occupation of father and mother

^a^: mediation analysis for adulthood education

^b^: mediation analysis for adulthood household income

## Discussion

This study found that CEPD was associated with adulthood suicidal ideation among the late adulthood group. This finding is partially consistent with previous studies reporting significant relationships between CEPD and mental health [9, 15]. For example, a cohort study of French elderly showed that those who experienced parental loss or separation during childhood were more likely to report new-onset of generalized anxiety disorder than those who did not [15].

However, our results are inconsistent with a prior study showing that the association between CEPD and suicide is significant only at < 50 years of age [11]. These different findings may reflect that our results are from Korea, a country that has achieved remarkably rapid progress in human development. For economic growth, gross domestic product per capita in Korea has risen from $60 in 1948 to $27,538 in 2016 [16]. Therefore, the social context experienced by people in Korea in late adulthood is different from those in early and middle adulthood. In our data, individuals in late adulthood were ~ 4-times more likely to suspend their education due to financial strain than individuals in early and middle adulthood (29.4% vs. 7.6%, respectively).

In addition, the causes of CEPD could differ between age groups. Individuals in late adulthood were ≥ 60 years old in 2011, so they were born prior to 1952. Thus, they went through the Korean War, which started in 1950. Casualties of the war could be therefore included under CEPD only among individuals in late adulthood. A cohort study in Nordic countries reported that all-cause mortality risk was higher among people who experienced parental death due to external causes than those whose parent died by natural death from diseases or medical conditions [17]. Therefore, the effect of CEPD on suicidal ideation among late adulthood may have been higher than the effect in early and middle adulthood.

Notably, our results suggest that adulthood household income may play a mediating role in a pathway linking CEPD to adulthood suicidal ideation, which was consistent with previous findings [12, 13]. For example, a study of Korean adults found that household income in adulthood could be a mediator in the pathway between childhood parental death and adulthood depressive symptoms [12]. According to life course theory, a sequence of linked exposures could influence later health problems, which means that lower SES resulting from CEPD could lead to mental health problems in adulthood [18]. Adolescents who experienced parental death might be more likely to attain lower education [19], and the low educational attainment could be linked to disparities in wealth and income. It has been found that lower SES could lead to a higher risk of mental health problems. For example, a cohort study analyzing the National Health Insurance Service-Senior claims data in Korea found that the poverty group had a higher risk of suicide than the high-income group among Korean adults aged 60 to 74 years old [20]. On the other hands, we should be cautious about interpreting the statistically non-significant results in the mediation analysis about education attainment. Compared to household income which indicates SES at the baseline of this study, educational attainment would reflect SES at least 30 years ago for the people in the late adulthood group when they were adolescents or early twenties.

This study showed that CEPD could be associated with suicidal ideation in late adulthood, and the relationship was mediated by adulthood household income. In Korea, the poverty rate among the elderly (> 65 years old) was 43.8% in 2017, which was the highest among OECD countries [21]. Also, Korea is known to have one of the highest suicide rates in the world, especially among the elderly. According to the Statistics Korea, suicide rate among ≥60 years old was more than 33 per 100,000 persons in 2019 and it was highest among all age groups [22]. Considering the high poverty rate and suicide rate in the Korean elderly, our research suggests that income security policy might be necessary to reduce suicide among the late adulthood group.

Some limitations should be noted in this study. First, we did not consider childhood economic status as childhood SES. Childhood economic status could be considered as a confounder in the association between CEPD and adulthood health, although some studies indicate that childhood economic status is a result of CEPD [19, 23]. Sub-analyses that included childhood economic status in the final model indicated results that are consistent with our findings. Second, surveys were conducted through in-person interviews, so there could be measurement error due to under-reporting of suicidal ideation because of concern about negative perception by the interviewer. Future studies should use administrative data. Third, we could not consider the cause of parental death. Because previous studies reported that depression and suicidal behavior could be influenced by the suicidal behaviors of a parent, future studies should consider the cause of parental death [24]. Fourth, there could be a possibility for selection bias due to missing data. We compared the distribution of covariates between our study population and those who were not included in the analysis due to missing data. The distribution was similar between two groups, but those with missing data were more likely to be the elderly and those whose mothers had lower educational attainment.

Nevertheless, this study has strengths, as it is the first study to examine the association between CEPD and adulthood suicidal ideation in Korea and the mediating role of adulthood SES on this relationship. Because we used a nationally representative large dataset, it is possible to examine the association between CEPD and adulthood suicidal ideation after excluding people who have experienced lifetime suicidal ideation.

## Conclusions

In summary, we found a significant association between CEPD and suicidal ideation in late adulthood using a nationally representative longitudinal dataset in Korea. Furthermore, the study reported that adulthood household income could mediate the pathway linking CEPD to suicidal ideation in late adulthood. The findings suggest that income security policy might be necessary to reduce suicide among the late adulthood group.

## Supplementary Information

**Additional file 1: Supplement 1.** Association between CEPD and adulthood suicidal ideation in South Korea (*N*=10,107). **Supplement 2.** Association between CEPD and adulthood suicidal ideation after adjusting for adulthood socio-economic status in South Korea (*N*=10,107). **Supplement 3.** The natural direct effects (NDE), natural indirect effects (NIE), and marginal total effects (MTE) expressed as risk ratios (ORs) of CEPD on adulthood suicidal ideation among the late adulthood group (*N*=3,779)

## Data Availability

The datasets analyzed during the current study are available in the Korea Institute for Health and Social Affairs, http://www.koweps.re.kr (28-Sep-2020).

## Abbreviations

CEPD: Childhood Experience of Parental Death
KOWEPS: Korea Welfare Panel Study
MTE: Marginal Total Effects
NDE: Natural Direct Effects
CDE: Controlled Direct Effects
NIE: Natural Indirect Effects
