# Supplementary material for: Role of SES on the association between childhood parental death and adulthood suicidal ideation: a mediation analysis using longitudinal dataset in South Korea
Source: BMC Psychiatry. 2021 Mar 23;21:162. doi: 10.1186/s12888-021-03146-w (PMC7986519; doi:10.1186/s12888-021-03146-w)
Supplement: Supplementary file 1 — Additional file 1: Supplement 1. Association between CEPD and adulthood suicidal ideation in South Korea (N=10,107). Supplement 2. Association between CEPD and adulthood suicidal ideation after adjusting for adulthood socio-economic status in South Korea (N=10,107). Supplement 3. The natural direct effects (NDE), natural indirect effects (NIE), and marginal total effects (MTE) expressed as risk ratios (ORs) of CEPD on adulthood suicidal ideation among the late adulthood group (N=3,779) [file 12888_2021_3146_MOESM1_ESM.docx]

**Additional file 1. Sub-analysis**

Sub-analysis was conducted to examine the association between CEPD and adulthood suicidal ideation and the mediating role of adulthood SES on the relationship when we included people who had a lifetime experience of suicidal ideation at baseline in the analyses.

Supplement 1. Association between CEPD and adulthood suicidal ideation in South Korea (N=10,107).

| CEPD |  | Suicidal ideation in 2012-2019 | | | | | | | |
| --- | --- | --- | --- | --- | --- | --- | --- | --- | --- |
|  |  | Early adulthood: 19-39 years old (N=2,999) | |  | Middle adulthood: 40-59 years old (N=3,329) | |  | Late adulthood: ≥60 years old (N=3,779) | |
|  |  | OR | 95% CI |  | OR | 95% CI |  | OR | 95% CI |
| No |  | 1.00 | Referent |  | 1.00 | Referent |  | 1.00 | Referent |
| Yes |  | 1.25 | (0.78–2.00) |  | 1.11 | (0.83–1.48) |  | 1.38** | (1.13–1.68) |

*P<0.05; **P<0.01; ***p<0.001

Adjusted for age, sex, education level of father and mother, and occupation of father and mother.

Supplement 2. Association between CEPD and adulthood suicidal ideation after adjusting for adulthood socio-economic status in South Korea (N=10,107).

| CEPD |  | Suicidal ideation in 2012-2019 | | | | | | | |
| --- | --- | --- | --- | --- | --- | --- | --- | --- | --- |
|  |  | Early adulthood: 19-39 years old (N=2,999) | |  | Middle adulthood: 40-59 years old (N=3,329) | |  | Late adulthood: ≥60 years old (N=3,779) | |
|  |  | OR | 95% CI |  | OR | 95% CI |  | OR | 95% CI |
| No |  | 1.00 | Referent |  | 1.00 | Referent |  | 1.00 | Referent |
| Yes |  | 1.13 | (0.70–1.83) |  | 1.09 | (0.81–1.46) |  | 1.33** | (1.09–1.63) |

*P<0.05; **P<0.01; ***p<0.001

Adjusted for age, sex, education level of father and mother, occupation of father and mother, education level, and household income.

Supplement 3. The natural direct effects (NDE), natural indirect effects (NIE), and marginal total effects (MTE) expressed as risk ratios (ORs) of CEPD on adulthood suicidal ideation among the late adulthood group (N=3,779)

| CEPD |  | Suicidal ideation in 2012-2019 | | | | | |
| --- | --- | --- | --- | --- | --- | --- | --- |
|  |  | NDE | | NIE | | MTE | |
|  |  | OR | 95% CI | OR | 95% CI | OR | 95% CI |
| Adulthood education attainment^a^ |  | 1.37** | (1.12– 1.67) | 1.00 | (0.80–1.26) | 1.38* | (1.02–1.86) |
| Adulthood household income^b^ |  | 1.32** | (1.08–1.61) | 1.06** | (1.02–1.09) | 1.40** | (1.14–1.71) |

*P<0.05; **P<0.01; ***p<0.001

Adjusted for age, sex, education level of father and mother, and occupation of father and mother.

a: mediation analysis for adulthood education

b: mediation analysis for adulthood household income
